# Dissecting tumor metabolic heterogeneity: Telomerase and large cell size metabolically define a sub-population of stem-like, mitochondrial-rich, cancer cells

**DOI:** 10.18632/oncotarget.5260

**Published:** 2015-08-27

**Authors:** Rebecca Lamb, Bela Ozsvari, Gloria Bonuccelli, Duncan L. Smith, Richard G. Pestell, Ubaldo E. Martinez-Outschoorn, Robert B. Clarke, Federica Sotgia, Michael P. Lisanti

**Affiliations:** ^1^ The Breast Cancer Now Research Unit, Institute of Cancer Sciences, University of Manchester, Manchester, UK; ^2^ The Manchester Centre for Cellular Metabolism (MCCM), Institute of Cancer Sciences, University of Manchester, Manchester, UK; ^3^ The Cancer Research UK Manchester Institute, University of Manchester, Manchester, UK; ^4^ The Sidney Kimmel Cancer Center, Philadelphia, PA, USA

**Keywords:** hTERT, telomerase, cell size, mitochondrial biogenesis, cancer stem cells, proteomic analysis, tumor metabolism

## Abstract

Tumor cell metabolic heterogeneity is thought to contribute to tumor recurrence, distant metastasis and chemo-resistance in cancer patients, driving poor clinical outcome. To better understand tumor metabolic heterogeneity, here we used the MCF7 breast cancer line as a model system to metabolically fractionate a cancer cell population. First, MCF7 cells were stably transfected with an hTERT-promoter construct driving GFP expression, as a surrogate marker of telomerase transcriptional activity. To enrich for immortal stem-like cancer cells, MCF7 cells expressing the highest levels of GFP (top 5%) were then isolated by FACS analysis. Notably, hTERT-GFP(+) MCF7 cells were significantly more efficient at forming mammospheres (i.e., stem cell activity) and showed increased mitochondrial mass and mitochondrial functional activity, all relative to hTERT-GFP(−) cells. Unbiased proteomics analysis of hTERT-GFP(+) MCF7 cells directly demonstrated the over-expression of 33 key mitochondrial proteins, 17 glycolytic enzymes, 34 ribosome-related proteins and 17 EMT markers, consistent with an anabolic cancer stem-like phenotype. Interestingly, MT-CO2 (cytochrome c oxidase subunit 2; Complex IV) expression was increased by >20-fold. As MT-CO2 is encoded by mt-DNA, this finding is indicative of increased mitochondrial biogenesis in hTERT-GFP(+) MCF7 cells. Importantly, most of these candidate biomarkers were transcriptionally over-expressed in human breast cancer epithelial cells *in vivo*. Similar results were obtained using cell size (forward/side scatter) to fractionate MCF7 cells. Larger stem-like cells also showed increased hTERT-GFP levels, as well as increased mitochondrial mass and function. Thus, this simple and rapid approach for the enrichment of immortal anabolic stem-like cancer cells will allow us and others to develop new prognostic biomarkers and novel anti-cancer therapies, by specifically and selectively targeting this metabolic sub-population of aggressive cancer cells. Based on our proteomics and functional analysis, FDA-approved inhibitors of protein synthesis and/or mitochondrial biogenesis, may represent novel treatment options for targeting these anabolic stem-like cancer cells.

## INTRODUCTION

Telomerase plays a central role both in the biology of normal aging, as well as in the development of human cancers [1, 2]. However, its exact role still remains poorly understood. Telomerase is a ribo-nucleoprotein, which contains both telomerase RNA (TERC) and telomerase reverse transcriptase (TERT) subunits [3]. Importantly, the over-expression of telomerase in both normal stem cells and cancer cells is sufficient to confer cell immortalization, such that these cells can divide beyond 50-70 divisions and bypass senescence [4–6].

Recently, Clarke and colleagues have taken advantage of the properties of human telomerase (hTERT), to enrich for a population of osteosarcoma cells with stem-like properties [7]. For this purpose, they fused a 1.5-kB fragment of the hTERT promoter to GFP, in order to select a sub-population of osteosarcoma cells with high-telomerase transcriptional activity by FACS analysis. More specifically, they demonstrated that these hTERT-high-activity osteosarcoma cells showed increased stem-like activity, anchorage-independent growth, invasiveness and metastatic activity, as well as chemo-therapy resistance [7]. As such, this hTERT-based approach for the purification of cancer stem-like cells (CSCs) has already been functionally validated.

Here, we have adapted this approach to the study of breast cancer stem-like cells, with a focus on proteomics analysis, biomarker discovery and cell metabolism. Importantly, we demonstrate that hTERT-high-activity breast cancer cells form mammospheres with higher efficiency, and show a proteomics profile consistent with an anabolic cancer stem cell phenotype. In support of this notion, we also show that hTERT-high-activity breast cancer cells have increased mitochondrial mass and activity, consistent with an increase in mitochondrial biogenesis.

## RESULTS

### Experimental approach: fluorescent enrichment of breast cancer stem cell activity

**Figure 1:**
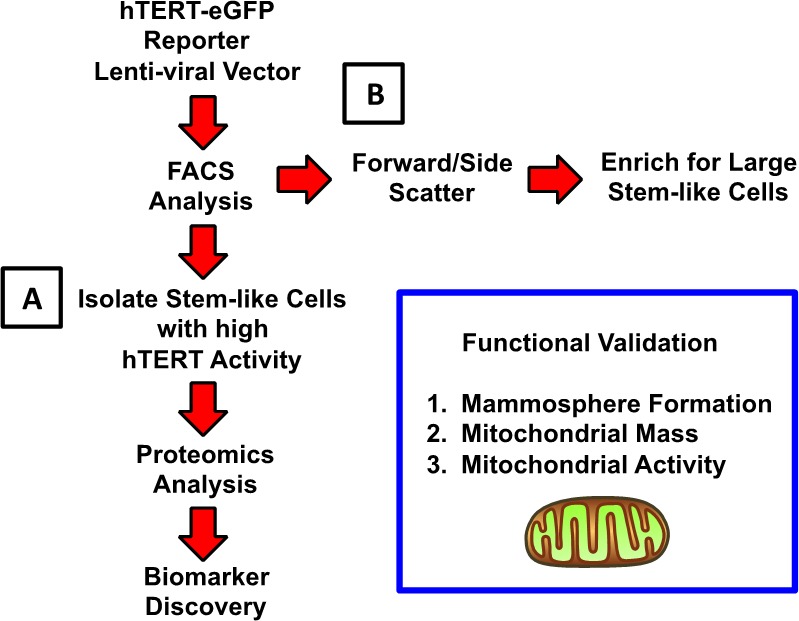
Summary of the overall experimental approach, using FACS analysis, to enrich for breast cancer stem-like cells: Focus on telomerase and cell size **A.** Telomerase Activity: To enrich for a population of immortal CSCs, we exploited a sensitive reporter system for the fluorescent detection of high telomerase transcriptional activity (hTERT-eGFP). We used this simplified approach to drive biomarker discovery, via unbiased label-free proteomics analysis. **B.** Cell Size (Forward Scatter/Side Scatter): Alternatively, we fractionated MCF7 cells based on forward/side scatter into larger and smaller cell populations. Previous studies using mouse mammary epithelial cells have demonstrated that stem-like cells can be enriched solely based on cell size [11]. For example, large stem-like cells with diameters >10 μm, defined by higher forward scatter during FACS analysis, showed a >4-fold increased ability to undergo mammosphere formation.

**Figure 2:**
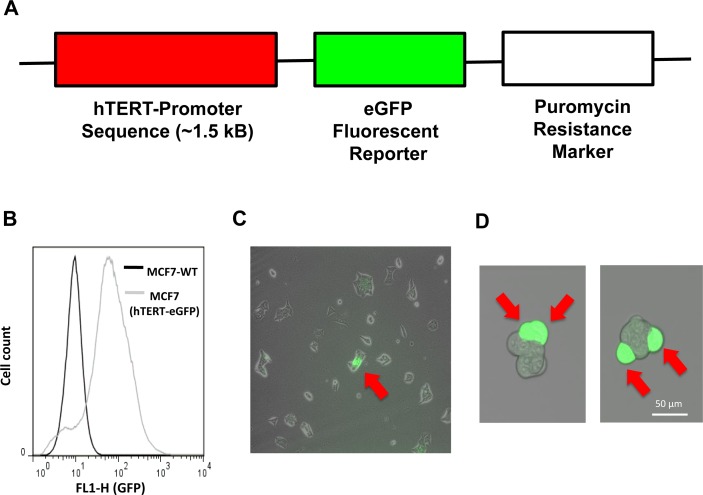
Generation of MCF7 cells harboring the hTERT-eGFP reporter, to select a sub-population of cells with high telomerase activity Panel **A.** MCF7 cells were transduced with a lentiviral vector driving eGFP protein expression, under the control of a 1.5-kB fragment of the hTERT promoter. This DNA construct also contains a puromycin-resistance cassette for antibiotic-resistance selection. Panel **B.** After selection with puromycin, MCF7-hTERT-eGFP cells were subjected to FACS sorting to visualize the broad distribution of eGFP expression, which serves as a surrogate marker of telomerase activity. Panel **C.** Note that very few cells in MCF7-hTERT-eGFP monolayers showed high GFP fluorescence. Panel **D.** Note the dramatic enrichment of GFP(+) cells in MCF7 cell mammospheres (> 50 μm), each containing usually 1-to-2 GFP-high cells.

To enrich for a population of immortal CSCs, we exploited a sensitive eGFP reporter system for the fluorescent detection of high telomerase transcriptional activity. Briefly, MCF7 cells were transduced with a lentiviral vector driving eGFP protein expression, under the control of a 1.5-kB fragment of the hTERT promoter. This DNA construct also contains a puromycin-resistance cassette for antibiotic-resistance selection. Schematic diagrams illustrating this overall experimental strategy and the construction of the hTERT-promoter-vector are shown in Figures 1 and 2.

After selection with puromycin, MCF7-hTERT-eGFP cells were subjected to FACS analysis to visualize the broad distribution of eGFP expression, which serves as a surrogate marker of telomerase activity. Importantly, fewer than 1 in a 100 cells in MCF7-hTERT-eGFP cell monolayers visually showed high GFP fluorescence. In striking contrast, there was a dramatic enrichment of GFP(+) cells in MCF7 cell mammospheres (> 50 μm), each containing usually 1-to-2 GFP-high cells (Figure 2).

To validate the enrichment of mammosphere forming cells, MCF7-hTERT-eGFP cells were fractionated into GFP-high (top 5%) and GFP-low (negative) groups. Then, five thousands cells from each group were seeded per well in 6-well low-attachment plates. These two groups were compared to the total unfractionated cell population.

**Figure 3:**
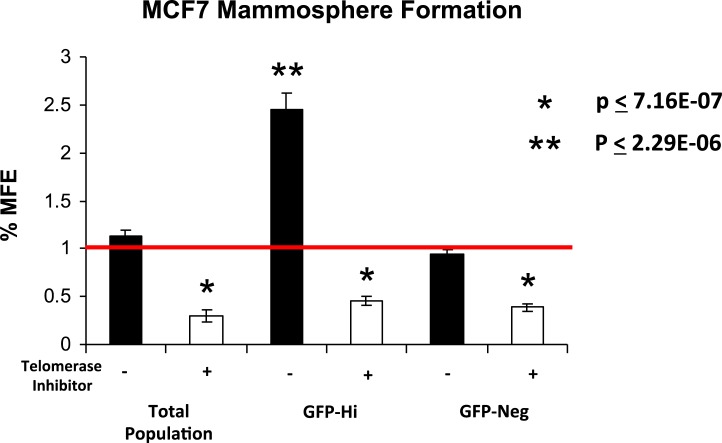
hTERT-eGFP-high MCF7 cells form mammospheres more efficiently, in a telomerase-dependent manner MCF7-hTERT-eGFP cells were fractionated into GFP-high (top 5%) and GFP-low (negative) groups. Then, five thousands cells from each group were seeded per well in 6-well low-attachment plates. These two groups were also compared to the total unfractionated cell population. Note that GFP-high cells form mammospheres with an efficiency nearly 2.5-fold greater than GFP-negative cells and the total unfractionated cell population. In this context, mammosphere formation was sensitive to that administration of a well-characterized telomerase inhibitor, namely MST-312, as expected. MFE, mammosphere forming efficiency.

Remarkably, Figure 3 shows that GFP-high cells form mammospheres with an efficiency nearly 2.5-fold greater than GFP-negative cells and the total unfractionated cell population. Importantly, in this context, mammosphere formation was sensitive to the administration of a well-characterized telomerase inhibitor, namely MST-312, as expected (Figure 3).

### Proteomics analysis of MCF7-hTERT-eGFP cells

In order to dissect the mechanism(s) by which high telomerase activity drives the survival and clonal expansion of CSCs, we next employed an unbiased label-free proteomics approach. The proteome of fractionated MCF7-hTERT-eGFP cells was directly determined, after FACS separation into GFP-high and GFP-low populations. For simplicity, we focused on the proteins that were over-expressed in GFP-high cells by > 1.5-fold. Our results are summarized in Tables 1, 2 and 3.

**Table 1:** Key Molecules Up-regulated in hTERT-GFP(+) MCF7 Cells Isolated by FACS: Mitochondria and Glycolysis

Symbol	Description	Fold-Upregulation (GPF(+)/GFP(−))	ANOVA
**Mitochondrial-Related Proteins (33)**			
MT-CO2	Cytochrome c oxidase subunit 2, mt-DNA encoded	20.48	7.27E-07
GRPEL1	GrpE protein homolog 1, mitochondrial	11.90	0.006
ECHS1	Enoyl-CoA hydratase, mitochondrial	8.64	9.13E-06
PARK7	Protein DJ-1	8.83	9.61E-07
TOMM7	Mitochondrial import receptor subunit TOM7	8.16	0.025
ATP5B	ATP synthase subunit beta, mitochondrial	7.76	1.81E-05
UQCRC2	Cytochrome b-c1 complex subunit 2, mitochondrial	6.75	2.34E-05
FASN	Fatty acid synthase	6.60	0.0002
IDH2	Isocitrate dehydrogenase [NADP] 2	5.77	6.95E-06
COX5A	Cytochrome c oxidase subunit 5A, mitochondrial	4.99	6.99E-06
ECH1	Delta(3,5)-Delta(2,4)-dienoyl-CoA isomerase, mitochondrial	4.95	0.0006
MCCC2	Methylcrotonoyl-CoA carboxylase beta chain, mitochondrial	4.84	2.55E-05
GATC	Glutamyl-tRNA(Gln) amidotransferase subunit C, mitochondrial	4.32	9.33E-06
ETFA	Electron transfer flavoprotein subunit alpha, mitochondrial	4.26	7.67E-05
HADH2	HSD17B10/HADH2; 3-hydroxyacyl-CoA dehydrogenase	4.26	0.0002
ACADVL	Very-long-chain specific acyl-CoA dehydrogenase, mitochondrial	4.06	0.0001
AK2	Adenylate kinase 2, mitochondrial	4.00	0.0001
IDH3A	Isocitrate dehydrogenase [NAD] subunit alpha, mitochondrial	3.83	5.05E-05
HADHA	Trifunctional enzyme subunit alpha, mitochondrial	3.43	2.89E-05
CS	Citrate synthase, mitochondrial	3.40	7.87E-06
HSPD1	60 kDa heat shock protein, mitochondrial	3.30	2.36E-05
DECR1	2,4-dienoyl-CoA reductase, mitochondrial	3.29	0.001
SLC25A10	Mitochondrial dicarboxylate carrier	3.24	0.0001
GSR	Glutathione reductase, mitochondrial	3.19	0.01
CYC1	Cytochrome c1, heme protein, mitochondrial	2.91	3.89E-06
NDUFA5	NADH dehydrogenase [ubiquinone] 1 alpha subcomplex subunit 5	2.83	5.39E-05
PRDX5	Peroxiredoxin-5, mitochondrial	2.62	0.0009
PRKDC	DNA-dependent protein kinase, catalytic subunit (maintains mt-DNA)	2.25	0.008
UQCR10	Cytochrome b-c1 complex subunit 9	2.22	0.037
SDHA	Succinate dehydrogenase complex, subunit A, flavoprotein	2.20	0.001
ABAT	4-aminobutyrate aminotransferase, mitochondrial	2.14	0.003
CYCS	Cytochrome c, somatic	2.13	9.35E-05
MRPL15	Mitochondrial ribosomal protein L15	2.11	0.002
**Glycolysis and Pentose Phosphate Pathway (PPP) Related Enzymes (17)**			
GAPDH	Glyceraldehyde-3-phosphate dehydrogenase	25.19	1.13E-05
PGAM1	Phosphoglycerate mutase 1 (Brain)	16.82	6.97E-06
G6PD	Glucose-6-phosphate 1-dehydrogenase	9.29	1.37E-06
ENO1	Alpha-enolase	9.08	1.08E-05
PKM2	Pyruvate kinase	8.15	0.0016
ALDOA	Fructose-bisphosphate aldolase	6.87	3.53E-06
HK2	Hexokinase-2	5.71	2.43E-06
GPI	Glucose-6-phosphate isomerase	5.61	4.43E-05
TALDO1	Transaldolase	5.20	0.00095
LDHA	L-lactate dehydrogenase A	4.65	2.82E-05
PGD	6-phosphogluconate dehydrogenase, decarboxylating	4.21	3.24E-05
PGK1	Phosphoglycerate kinase 1	3.97	0.01
HK3	Hexokinase-3	3.66	0.0003
PFKP	6-phosphofructokinase type C	3.48	0.0002
PGAM2	Phosphoglycerate mutase 2	3.38	0.006
FBP1	Fructose-1,6-bisphosphatase 1	2.72	0.003
TKT	Transketolase	2.15	0.0003

**Table 2:** Key Molecules Up-regulated in hTERT-GFP(+) MCF7 Cells Isolated by FACS: the EMT

Symbol	Description	Fold-Upregulation (GPF(+)/GFP(−))	ANOVA
**EMT-Related Marker Proteins (17)**			
MTPN	Myotrophin	41.58	0.0016
KRT19	Keratin, type I cytoskeletal 19	15.04	9.00E-06
ACTR3	Actin-related protein 3	12.74	0.0002
FLNB	Filamin-B	9.30	3.50E-08
GSN	Gelsolin	9.07	4.58E-05
ACTN4	Alpha-actinin-4	6.63	0.0001
ACTA2	Actin, aortic smooth muscle	5.91	0.0003
ACTN1	Alpha-actinin-1	5.65	0.0004
CKAP4	Cytoskeleton-associated protein 4	3.86	9.86E-05
MYO5C	Unconventional myosin-Vc	3.84	0.0002
SPTAN1	Spectrin alpha chain, non-erythrocytic 1	3.81	0.0002
TAGLN2	Transgelin-2	3.55	4.57E-05
CD44	CD44 antigen	3.20	0.015
MYOF	Myoferlin	2.47	0.0007
MYH14	Myosin-14	2.24	0.02
PFN2	Profilin	2.21	0.0002
SPTBN1	Spectrin beta non-erythrocytic 1	2.15	0.0017
**Markers of Proliferation (3)**			
PCNA	Proliferating cell nuclear antigen	6.50	2.45E-05
MCM3	MCM3 mini-chromosome maintenance deficient 3	4.05	0.0005
PA2G4	Proliferation-associated protein 2G4	3.29	0.01
**Anti-Oxidant Proteins (2)**			
NQO1	NAD(P)H dehydrogenase, quinone 1	6.43	3.90E-05
SOD1	Superoxide dismutase [Cu-Zn]	4.49	2.13E-07

**Table 3:** Key Molecules Up-regulated in hTERT-GFP(+) MCF7 Cells Isolated by FACS: Protein Synthesis and Chaperones

Symbol	Description	Fold-Upregulation (GPF(+)/GFP(−))	ANOVA
**Chaperones for Protein Folding (10)**			
PPIA	Peptidyl-prolyl cis-trans isomerase A	Infinity	0.007
HSPA2	Heat shock-related 70kDa protein 2	27.53	0.003
HSPA1L	Heat shock 70 kDa protein 1-like	10.66	0.01
HSPA1B	Heat shock 70kDa protein 1A	9.65	0.0007
HSP90AB1	Heat shock protein HSP 90-beta	6.37	5.08E-06
HSP90B1	Heat shock protein Grp94	6.13	0.036
HSPA8	Heat shock cognate 71 kDa protein	4.40	0.0003
HSPD1	60 kDa heat shock protein, mitochondrial	3.30	2.36E-05
HSPA4	Heat shock 70kDa protein 4	2.90	4.57E-05
FKBP4	Peptidyl-prolyl cis-trans isomerase	2.21	0.003
**Ribosomal Proteins (10)**			
RPS15	40S ribosomal protein S15	6.70	1.08E-05
RPS3A	40S ribosomal protein S3A	4.78	3.87E-06
RPS4X	40S ribosomal protein S4, X-linked isoform	4.16	0.00075
RPL11	60S ribosomal protein L11	3.55	0.0001
RPL7	60S ribosomal protein L7	3.18	0.0001
RPS2	40S ribosomal protein S2	2.91	0.00016
RPS5	40S ribosomal protein S5	2.53	0.0002
RPL15	60S ribosomal protein L15	2.28	0.004
RPL32	60S ribosomal protein L32	2.03	0.0008
RPSA	40S ribosomal protein SA	2.00	0.018
**Translation initiation factors (5)**			
EIF3F	Eukaryotic translation initiation factor 3 subunit F	6.12	0.00016
EIF5A	Eukaryotic translation initiation factor 5A-1	3.36	0.0002
EIF3S9	Eukaryotic translation initiation factor 3 subunit B	2.17	0.0009
EIF4A2	Eukaryotic initiation factor 4A-II	2.06	0.001
EIF2S3	Eukaryotic translation initiation factor 2, subunit 3 gamma, 52kDa	1.88	0.003
**Elongation factors (4)**			
EEF1G	Elongation factor 1-gamma	14.43	5.54E-07
EEF1A1	Elongation factor 1 alpha 1	4.77	1.42E-05
EEF2	Elongation factor 2	2.53	0.0003
EEF1B2	Elongation factor 1-beta	2.18	0.001
**Enzymes for tRNA synthesis (5)**			
GATC	Glutamyl-tRNA(Gln) amidotransferase subunit C, mitochondrial	4.32	9.33E-06
AARS	Alanyl-tRNA synthetase	4.16	7.58E-05
FARSB	Phenylalanine--tRNA ligase beta subunit	2.63	0.009
EPRS	Bifunctional glutamate/proline--tRNA ligase	2.00	0.01
KARS	Lysine--tRNA ligase	1.95	0.007

Importantly, >30 mitochondrial-related proteins were over-expressed in GFP-high cells (Tables 1). Most of these proteins were related to oxidative phosphorylation, the TCA cycle, or mitochondrial biogenesis. Remarkably, MT-CO2 (a mt-DNA encoded protein) was upregulated by >20-fold, reflecting a significant increase in mitochondrial biogenesis. Consistent with an overall anabolic phenotype, 17 enzymes related to glycolysis and the pentose phosphate pathway were also upregulated in GFP-high cells (Table 1).

CSCs undergo an EMT, which facilitates cell migration, invasion and metastatic dissemination [8]. Thus, we also examined the expression of EMT markers, and cytoskeletal proteins associated with cell migration (Table 2). Greater than 17 proteins known to be associated with this EMT phenotype were upregulated in GFP-high cells. Such examples include myotrophin (>40-fold), keratin-19 (>15-fold), smooth muscle actin (>5-fold), and CD44 (>3-fold). Markers of proliferation (PCNA; >6-fold), and the anti-oxidant response (NQO1; >6-fold) were also significantly increased.

Elevated protein synthesis is another important feature of the anabolic CSC phenotype [9]. As predicted, GFP-high cells show the upregulation of >30 proteins related to protein synthesis (Table 3), including protein folding chaperones, ribosome-related proteins, translation initiation factors, peptide elongation factors, as well as enzymes for tRNA synthesis.

Taken together, hTERT-eGFP-high cells over-express >100 proteins related to an anabolic CSC phenotype.

### Relevance of hTERT targets in human breast cancers cells *in vivo*

To determine the clinical relevance of our findings, we next assessed whether the hTERT proteomic targets that we identified in GFP-high cells were transcriptionally over-expressed, in human breast cancer cells *in vivo*. For this purpose, we exploited a clinical data set of tumor samples from *N* = 28 breast cancer patients. These tumor samples were subjected to laser-capture micro-dissection, to separate epithelial cancer cells from adjacent tumor stroma [10]. Overall, greater than seventy hTERT targets (related to mitochondria, glycolysis, the EMT, and protein synthesis) that we identified in GFP-high cells were also transcriptionally elevated in human breast cancer cells *in vivo*. Tables 4, 5 and 6 present a detailed summary of these findings. These new hTERT protein targets that we identified in MCF7-hTERT-eGFP cells may be especially relevant for improving human breast cancer diagnosis and therapy.

**Table 4:** hTERT Protein Targets Transcriptionally Up-regulated in Breast Cancer: Focus on Mitochondria and Glycolysis

Symbol	Gene Description	Up-regulation (fold-change)	P-value
**Mitochondrial-Related Proteins (23)**			
MCCC2	Methylcrotonoyl-CoA carboxylase beta chain, mitochondrial	5.48	5.78E-07
ATP5B	ATP synthase subunit beta, mitochondrial	5.04	2.75E-06
UQCRC2	Cytochrome b-c1 complex subunit 2, mitochondrial	4.84	5.73E-06
PARK7	Protein DJ-1	4.26	4.08E-05
ECHS1	Enoyl-CoA hydratase, mitochondrial	4.05	8.22E-05
COX5A	Cytochrome c oxidase subunit 5A, mitochondrial	3.62	3.22E-04
HSPD1	60 kDa heat shock protein, mitochondrial	3.42	5.93E-04
DECR1	2,4-dienoyl-CoA reductase, mitochondrial	3.38	6.86E-04
HADHA	Trifunctional enzyme subunit alpha, mitochondrial	3.27	9.34E-04
CYC1	Cytochrome c1, heme protein, mitochondrial	3.08	1.64E-03
TOMM7	Mitochondrial import receptor subunit TOM7	3.03	1.85E-03
CYCS	Cytochrome c, somatic	2.92	2.52E-03
NDUFA5	NADH dehydrogenase [ubiquinone] 1 alpha subcomplex subunit 5	2.75	4.07E-03
CS	Citrate synthase, mitochondrial	2.66	5.13E-03
IDH2	Isocitrate dehydrogenase [NADP] 2	2.46	8.55E-03
GRPEL1	GrpE protein homolog 1, mitochondrial	2.39	1.01E-02
MRPL15	Mitochondrial ribosomal protein L15	2.26	1.39E-02
AK2	Adenylate kinase 2, mitochondrial	2.20	1.59E-02
IDH3A	Isocitrate dehydrogenase [NAD] subunit alpha, mitochondrial	2.16	1.78E-02
PRKDC	DNA-dependent protein kinase, catalytic subunit (maintains mt-DNA)	2.14	1.85E-02
ABAT	4-aminobutyrate aminotransferase, mitochondrial	2.08	2.14E-02
ECH1	Delta(3,5)-Delta(2,4)-dienoyl-CoA isomerase, mitochondrial	1.97	2.72E-02
ETFA	Electron transfer flavoprotein subunit alpha, mitochondrial	1.75	4.25E-02
**Glycolysis and Pentose Phosphate Pathway (PPP) Related Enzymes (11)**			
TALDO1	Transaldolase	4.13	6.35E-05
ALDOA	Fructose-bisphosphate aldolase	3.60	3.45E-04
GPI	Glucose-6-phosphate isomerase	3.36	7.28E-04
FBP1	Fructose-1,6-bisphosphatase 1	3.35	7.47E-04
PKM2	Pyruvate kinase	3.26	9.79E-04
GAPDH	Glyceraldehyde-3-phosphate dehydrogenase	2.97	2.22E-03
PGK1	Phosphoglycerate kinase 1	2.46	8.66E-03
PGAM1	Phosphoglycerate mutase 1 (Brain)	2.55	6.87E-03
LDHA	L-lactate dehydrogenase A	2.42	9.42E-03
TKT	Transketolase	2.20	1.60E-02
ENO1	Alpha-enolase	1.96	2.75E-02

**-Transcriptional profiling data derived from the analysis of N=28 breast cancer patients are shown, high-lighting the levels of fold-upregulation observed in the epithelial cancer cell compartment (relative to the tumor stroma), and corresponding p-values derived from the analysis of these clinical samples.**

**Table 5:** hTERT Protein Targets Transcriptionally Up-regulated in Breast Cancer: Focus on the EMT

Symbol	Gene Description	Up-regulation (fold-change)	P-value
**EMT-Related Marker Proteins (11)**			
FLNB	Filamin-B	4.81	6.21E-06
KRT19	Keratin, type I cytoskeletal 19	4.39	2.66E-05
SPTAN1	Spectrin alpha chain, non-erythrocytic 1	4.19	5.16E-05
MYO5C	Unconventional myosin-Vc	3.79	1.90E-04
CD44	CD44 antigen	3.44	5.69E-04
ACTR3	Actin-related protein 3	3.15	1.35E-03
MYOF	Myoferlin	2.67	5.00E-03
TAGLN2	Transgelin-2	2.42	9.47E-03
PFN2	Profilin	2.16	1.78E-02
ACTN4	Alpha-actinin-4	2.12	1.94E-02
CKAP4	Cytoskeleton-associated protein 4	1.88	3.29E-02
**Markers of Proliferation (1)**			
PCNA	Proliferating cell nuclear antigen	3.58	3.64E-04
**Anti-Oxidant Proteins (2)**			
SOD1	Superoxide dismutase [Cu-Zn]	5.37	8.58E-07
NQO1	NAD(P)H dehydrogenase, quinone 1	3.49	4.81E-04

**-Transcriptional profiling data derived from the analysis of N=28 breast cancer patients are shown, high-lighting the levels of fold-upregulation observed in the epithelial cancer cell compartment (relative to the tumor stroma), and corresponding p-values derived from the analysis of these clinical samples.**

**Table 6:** hTERT Protein Targets Transcriptionally Up-regulated in Breast Cancer: Focus on Protein Synthesis and Chaperones

Symbol	Gene Description	Up-regulation (fold-change)	P-value
**Chaperones for Protein Folding (8)**			
FKBP4	Peptidyl-prolyl cis-trans isomerase	5.02	2.95E-06
HSP90AB1	Heat shock protein HSP 90-beta	4.93	4.03E-06
PPIA	Peptidyl-prolyl cis-trans isomerase A	4.29	3.74E-05
HSPA4	Heat shock 70kDa protein 4	3.75	2.18E-04
HSPD1	60 kDa heat shock protein, mitochondrial	3.42	5.93E-04
HSPA8	Heat shock cognate 71 kDa protein	2.54	7.06E-03
HSP90B1	Heat shock protein Grp94	2.43	9.33E-03
HSPA1B	Heat shock 70kDa protein 1A	1.56	6.29E-02
**Ribosomal Proteins (10)**			
RPL7	60S ribosomal protein L7	5.21	1.53E-06
RPS2	40S ribosomal protein S2	4.77	7.21E-06
RPL15	60S ribosomal protein L15	4.60	1.28E-05
RPS3A	40S ribosomal protein S3A	4.59	1.35E-05
RPS5	40S ribosomal protein S5	4.41	2.45E-05
RPL32	60S ribosomal protein L32	4.03	8.74E-05
RPS4X	40S ribosomal protein S4, X-linked isoform	3.92	1.27E-04
RPS15	40S ribosomal protein S15	3.72	2.41E-04
RPL11	60S ribosomal protein L11	2.74	4.16E-03
**Translation initiation factors (2)**			
EIF3F	Eukaryotic translation initiation factor 3 subunit F	5.07	2.48E-06
EIF4A2	Eukaryotic initiation factor 4A-II	2.68	4.83E-03
**Elongation factors (4)**			
EEF1B2	Elongation factor 1-beta	4.08	7.56E-05
EEF2	Elongation factor 2	4.01	9.29E-05
EEF1G	Elongation factor 1-gamma	3.71	2.44E-04
EEF1A1	Elongation factor 1 alpha 1	3.16	1.30E-03
**Enzymes for tRNA synthesis (5)**			
EPRS	Bifunctional glutamate/proline--tRNA ligase	4.06	8.10E-05
KARS	Lysine--tRNA ligase	2.81	3.49E-03

**-Transcriptional profiling data derived from the analysis of N=28 breast cancer patients are shown, high-lighting the levels of fold-upregulation observed in the epithelial cancer cell compartment (relative to the tumor stroma), and corresponding p-values derived from the analysis of these clinical samples.**

### hTERT-eGFP-high MCF7 cells show an increase in mitochondrial mass and functional activity

**Figure 4:**
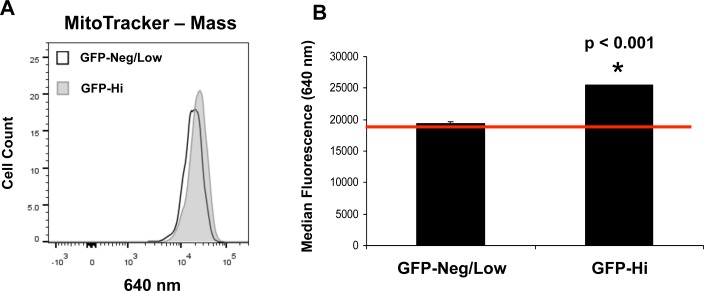
hTERT-eGFP-high MCF7 cells show an increase in mitochondrial mass Panel **A.** Note that as compared to GFP-low cells (bottom 5%), GFP-high cells (top 5%) demonstrate a significant shift to the right, for mitochondrial mass (MitoTracker Deep-Red probe). Panel **B.** FACS quantification of median fluorescence intensity is presented. As such, increased mitochondrial mass (1.3-fold) may be a critical element of the anabolic TIC phenotype. *p* < 0.001.

**Figure 5:**
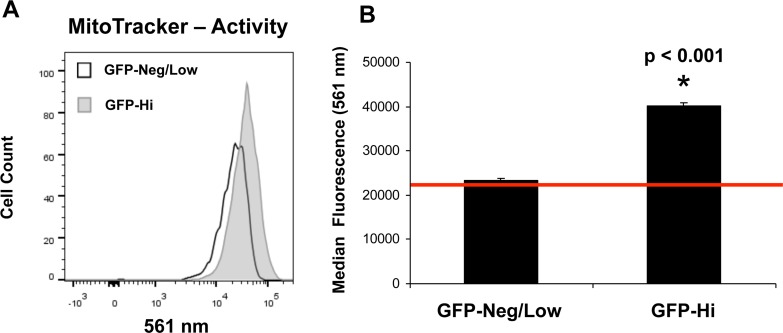
hTERT-eGFP-high MCF7 cells show an increase in mitochondrial activity Panel **A.** Note that as compared to GFP-low cells (bottom 5%), GFP-high cells (top 5%) demonstrate a significant shift to the right, for mitochondrial membrane potential (MitoTracker Orange probe). Panel B: FACS quantification of median fluorescence intensity is presented, representing a 1.7-fold increase. *p* < 0.001.

To directly validate the mitochondrial phenotype of hTERT-eGFP-high cells (top 5%), we used two well-established fluorescent probes to quantitate mitochondrial membrane potential and mass, by FACS analysis. More specifically, we used MitoTracker Orange (561-nm) as a reporter for mitochondrial membrane potential and MitoTracker Deep-Red (640-nm) as a marker of mitochondrial mass. Importantly, Figures 4 and 5 show that as compared to GFP-low cells (bottom 5%), GFP-high cells (top 5%) demonstrate a significant shift to the right, for both mass and membrane potential. FACS quantitation of median fluorescence intensity shows that both of these mitochondrial-related measurements are significantly elevated in GFP-high cells. As such, mitochondrial mass and function may be critical elements of the anabolic TIC phenotype.

### Using large cell size to enrich telomerase activity and mitochondrial mass

Previous studies using mouse mammary epithelial cells have demonstrated that stem-like cells can be enriched solely based on cell size [11]. For example, large stem-like cells with diameters >10 μm, defined by higher forward scatter during FACS analysis, showed a >4-fold increased ability to undergo 3-D mammosphere formation. Moreover, these large stem-like mammary cells also had the ability to efficiently repopulate and regenerate the mammary gland *in vivo* [11].

**Figure 6:**
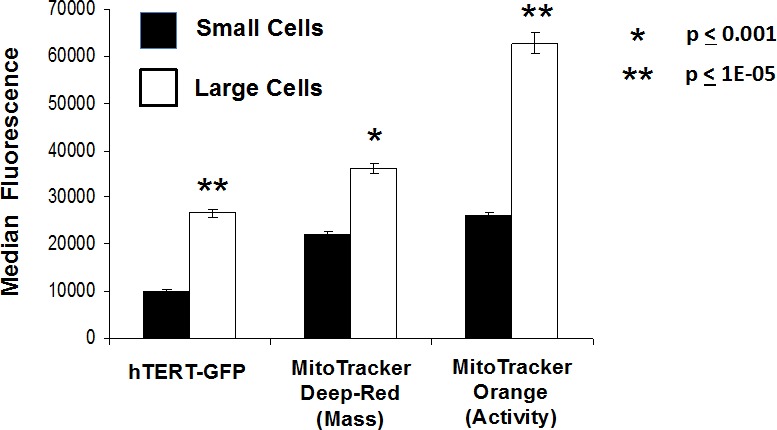
Fractionation of hTERT-eGFP MCF7 cells by cell size allows the separation of larger and smaller cell sub-populations, with distinct metabolic functional properties We fractionated MCF7-hTERT-eGFP cells based on forward/side scatter into larger and smaller cell populations. Note that larger MCF7 cells showed a 2.65-fold increase in hTERT-eGFP fluorescence, as compared with the smaller cell population. Similarly, larger cells also showed a 1.6-fold increase in mitochondrial mass (MitoTracker Deep-Red) and a 2.4-fold increase in mitochondrial activity (membrane potential), as measured using MitoTracker Orange. Thus, larger cell size directly correlates with telomerase activity and mitochondrial mass/activity, which would be consistent with an anabolic CSC phenotype.

Thus, here we fractionated MCF7-hTERT-eGFP cells by size, based on forward/side scatter, into two populations: i) *larger cells* (∼15% of the total population) and ii) *smaller cells* (∼85% of the total population) (Figure 6). Interestingly, larger MCF7 cells showed a 2.65-fold increase in hTERT-eGFP fluorescence, as compared with the smaller cell population. Importantly, larger cells also showed a 1.6-fold increase in mitochondrial mass (MitoTracker Deep-Red) and a 2.4-fold increase in mitochondrial activity (membrane potential), as measured using MitoTracker Orange (Figure 6).

As such, larger cell size in MCF7 cells directly correlates with telomerase activity (cell immortalization) and mitochondrial mass/activity, which would be consistent with an anabolic CSC phenotype. These results provide independent validation for the idea that high hTERT activity (“stemness”) is functionally associated with increased mitochondrial mass and activity in breast cancer cells, and co-segregates with large cell size. Importantly, large cell size is determined by increased PI3K/AKT/mTOR-signaling, which drives significant increases in overall protein synthesis [12–14]. This finding is consistent with our results from proteomics analysis, showing an increase in the abundance of the protein synthesis machinery (See Tables 3 and 6).

## DISCUSSION

Here, we have used an hTERT-promoter-eGFP-reporter system to identify and purify a sub-population of MCF7 cells, with high hTERT transcriptional activity, by FACS analysis. These hTERT-eGFP-high cells formed mammospheres with greater efficiency, as predicted, consistent with the idea that this sub-population of cells is enriched in cancer stem-like cells. Importantly, proteomics analysis of these hTERT-eGFP-high MCF7 cells revealed the upregulation of mitochondrial proteins, glycolytic enzymes and EMT markers, as well as components of the protein synthesis machinery, such as ribosome-related proteins and chaperones for protein folding. Interestingly, MT-CO2 (cytochrome c oxidase subunit 2; Complex IV) expression was increased by >20-fold. As MT-CO2 is encoded by mt-DNA, this finding is indicative of increased mitochondrial biogenesis in hTERT-eGFP-high MCF7 cells. We then functionally validated that hTERT-eGFP-high MCF7 cells show increases in mitochondrial mass and activity, using two distinct MitoTracker probes. Complementary results were obtained using cell size to fractionate MCF7 cells. Larger stem-like cells showed increased hTERT-GFP levels, as well as increased mitochondrial mass and function. Thus, these two independent approaches for the enrichment of immortal anabolic CSCs should allow the development of new prognostic biomarkers and related novel anti-cancer therapies.

**Figure 7:**
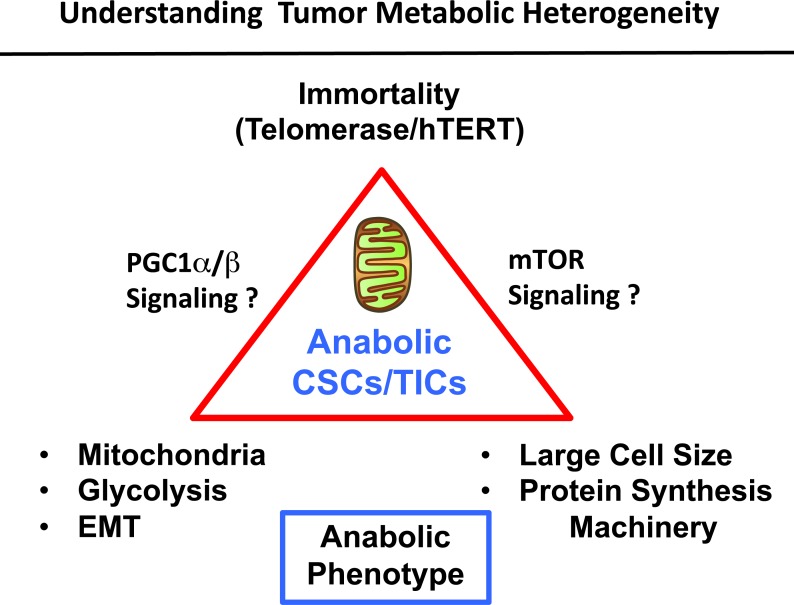
Understanding the role of immortality, anabolic metabolism and cell size in stem-like cancer cells Here, we used FACS analysis to begin to dissect metabolic heterogeneity in tumor cells. More specifically, we showed that anabolic stem-like cancer cells can be purified using hTERT-eGFP as a surrogate marker of telomerase activity. These eGFP-high cells showed increased stem cell activity (mammosphere formation), as well as functional increases in mitochondrial mass and activity. Further studies with unbiased label-free proteomics revealed the upregulation of mitochondrial proteins, glycolytic enzymes and EMT markers, as well as ribosome subunits and other components of the proteins synthesis machinery. These proteomic studies are consistent with, and support an anabolic stem-like cancer cell phenotype. Quantitatively similar results were obtained using large cell size to purify anabolic stem-like cancer cells. We speculate that high telomerase activity drives an increase in mitochondrial power, via positive regulation by PGC1-α/β. Moreover, we suggest that increased mTOR signaling may contribute to larger cell size, via increased protein synthesis. The PI3K/AKT/mTOR pathway is known to i) control cell size by positively regulating protein synthesis and ii) telomerase (hTERT) forms a physical complex with mTOR and can therefore regulate its activity. In summary, our results metabolically define a sub-population of stem-like, mitochondrial-rich, cancer cells, allowing us to understand the possible origins of metabolic heterogeneity in human tumors.

Interestingly, recent studies in aging and cancer have both directly linked telomerase activity to mitochondrial function, via the hTERT-p53-PGC1 signaling axis [15–18]. More specifically, telomerase is required for the proper expression of PGC1-α/β, a major nuclear-encoded mitochondrial transcription factor. Studies in aging demonstrate that a loss of telomerase functionally impairs mitochondria, via the induction of p53 which down-regulates PGC1-α/β expression [16]. Conversely, during tumorigenesis, high telomerase expression helps to promote cancer progression, by reducing p53 and, therefore, elevating PGC1-α/β expression [19–21]. Taken together, these mechanistic studies are consistent with our current findings, in which we link telomerase transcriptional activity in cancer stem cells to mitochondrial biogenesis, both functionally and via proteomics analysis (Figure 7).

Cell size is normally controlled by the PI3K/AKT/mTOR-pathway, which converges on protein synthesis, via S6-kinase signaling [12]. Therefore, large cell size is known to be associated with increased protein synthesis [13, 14]. Interestingly, telomerase (hTERT) forms a physical complex with mTOR and can therefore regulate its functional activity [22, 23]. Consistent with this hypothesis, we demonstrate here that stem-like anabolic cancer cells, with high-telomerase transcriptional activity, also show remarkable increases in i) *protein synthesis machinery* (chaperones, ribosome-related proteins, translation initiation factors, peptide elongation factors, enzymes for tRNA synthesis) and ii) are associated with *large cell size* (Figure 7).

Interestingly, large cell size is also a conserved characteristic of circulating tumor cells (CTCs), which share several properties with CSCs or TICs [24–26]. For example, CTCs – like CSCs – are found in clusters, are associated with distant metastasis, and can initiate tumor formation in xenografted mice [27]. Also, recent studies demonstrate that CTCs show an increase in mitochondrial mass and OXPHOS, most likely reflecting increased mitochondrial biogenesis, secondary to elevated levels of PGC1-α/β [28].

We recently directly compared the proteome of MCF7 cell monolayers to MCF7-derived mammospheres, using label-free unbiased proteomics analysis. Consistent with our current findings, this analysis demonstrated that MCF7-derived mammospheres show the over-expression of >60 mitochondrial-related proteins and >80 components of the protein synthesis machinery [9, 29]. Complementary results were also obtained with MCF7 cells transfected with FOXM1, a key stem cell associated transcription factor that is a downstream target of the Wnt/β-catenin signaling pathway [30]. Interestingly, MCF7-FOXM1 cells showed a >3-fold increase in mammosphere formation, as compared with empty-vector alone control cells. Thus, several independent proteomic data sets now show the conserved up-regulation of both i) mitochondrial biogenesis and ii) protein synthesis, as key elements of the anabolic CSC phenotype.

Importantly, functional validation studies revealed that mammosphere formation could be efficiently blocked with well-established inhibitors of mitochondrial function and/or inhibitors of protein synthesis, such as oligomycin A and puromycin, respectively [9, 29]. Moreover, FDA-approved drugs which inhibit mitochondrial biogenesis or OXPHOS as a manageable side-effect (azithromycin, doxycycline and pyrvinium pamoate) also efficiently blocked mammosphere formation [31]; an FDA-approved inhibitor of protein synthesis, namely rapamycin, inhibited mammosphere formation as well [9]. Remarkably, the growth of mammosphere cultures derived from primary breast cancer cells (isolated from metastatic disease sites) was sensitive to inhibition with doxycycline [32]. Thus, these diverse classes of FDA-approved drugs should all be considered for testing in future phase II clinical trials, such as window-of-opportunity trials. In direct support of this idea, everolimus (Afinitor; a rapamycin-analogue and inhibitor of protein synthesis) has already shown significant efficacy in clinical trials in breast cancer patients [33].

In summary, we show here that high telomerase activity metabolically defines a sub-population of anabolic CSCs, with increased mitochondrial mass and large cell size. Overall, greater than seventy hTERT targets (related to mitochondria, glycolysis, the EMT, and protein synthesis) that we identified in hTERT-eGFP-high cells were also transcriptionally elevated in human breast cancer cells *in vivo*. Thus, the new hTERT metabolic targets that we identified here may be important for improving human breast cancer diagnosis and therapy.

## MATERIALS AND METHODS

### Materials

MCF7 cells were purchased from the ATCC. Gibco-brand cell culture media (DMEM and DMEM/F12) was purchased from Life Technologies. The lentiviral vector encoding the hTERT promoter linked to eGFP was custom-made by Genecopoeia (USA). MitoTracker probes (Deep Red and Orange) were purchased from Molecular Probes/Invitrogen, via Life Technologies. The telomerase inhibitor IX (MST-312; sc-204333) was obtained commercially from Santa Cruz Biotech (USA).

### Construction of the hTERT-eGFP lentiviral vector

The following 1.5 kB sequence was used as the hTERT-promoter to generate the hTERT-eGFP-Puro^R^ cassette, and cloned into a lentiviral vector, custom-made by Genecopoeia: 5′-TAAAATTGTGTTTTCTATGTTGGCTTCTCTGCAGAGAACCAGTGTAAGCTACAACTTAACTTTTGTTGGAACAAATTTTCCAAACCGCCCCTTTGCCCTAGTGCAGAGACAATTCACAAACACAGCCCTTTAAAAAGGCTTAGGGATCACTAAGGGGATTTCTAGAAGAGCGACCTGTAATCCTAAGTATTTACAAGACGAGGCTAACCTCCAGGAGCGTGACAGCCCAGGGAGGGTGCGAGGCCTGTTCAAATGCTAGCTCCATAAATAAAGCAATTTCCTCC GGCAGTTTCTGAAAGTAGGAAAGGTTACATTTAAGGTTGCGTTTGTTAGCATTTCAGTGTTTGCCGACCTCAGCTACAGCATCCCTGCAAGGCCTCGGGAGACCCAGAAGTTTCTCGCCCCTTAGATCCAAACTTGAGC AACCCGGAGTCTGGATTCCTGGGAAGTCCTAGCTGTCCTGCGGTTGTGCCGGGGCCCCAGGTCTGGAGGGGACCAGTGGCCGTGTGGCTTCTACTGCTGGGCTGGAAGTCGGGCCTCCTAGCTCTGCAGTCCGAGGCTTGGAGCCAGGTGCCTGGACCCCGAGGTTGCCCTCCACCCTGTGCGGGCGGGATGTGACCAGATGTTGGCCTCATCTGCCAGACAGAGTGCCG GGGCCCAGGGTCAAGGCCGTTGTGGCTGGTGTGAGGCGCCCGGTGCGCGGCCAGCAGGAGCGCCTGGCTCCATTTCCCACCCTTTCTCGACGGGACCGCCCCGGTGGGTGATTAACAGATTTGGGGTGG TTTGCTCATGGTGGGGACCCCTCGCCGCCTGAGAACCTGCAAAGAGAAATGACGGGCCTGTGTCAAGGAGCCCAAGTCGCGGGGAAGTGTTGCAGGGAGGCACTCCGGGAGGTCCCGCGTGCCCGTCC AGGGAGCAATGCGTCCTCGGGTTCGTCCCCAGCCGCGTCTACGCGCCTCCGTCCTCCCCTTCACGTCCGGCATTCGTGGTGCCCGGAGCCCGACGCCCCGCGTCCGGACCTGGAGGCAGCCCTGGGTCTCCGGATCAGGCCAGCGGCCAAAGGGTCGCCGCACGCACCTGTTCCCAGGGCCTCCACATCATGGCCCCTCCCTCGGGTTACCCCACAGCCTAGGCCGATTCGACCTCTCTCCGCTGGGGCCCTCGCTGGCGTCCCTGCACCCTGGGAGCGCGAGCGGCGCGCGGGCGGGGAAGCGCGGCCCAGACCCCCGGGTCCGCCCGGAGCAGCTGCG CTGTCGGGGCCAGGCCGGGCTCCCAGTGGATTCGCGGGCACAGACGCCCAGGACCGCGCTTCCCACGTGGCGGAGGGACTGGGGACCCGGGCACCCGTCCTGCCCCTTCACCTTCCAGCTCCGCCTCCTCCGCGCGGACCCCGCCCCGTCCCGACCCCTCCCGGGTCCCCGGCCCAGCCCCCTCCGGGCCCTCCCAGCCCCTCCCCTTCCTTTCCGCGGCCCCGCCCTCTCCTCGCGGCGCGAGTT-3′.

### Viral transduction and cell selection

Lentiviral particles harboring the hTERT-eGFP-Puro^R^ cassette were prepared and used to stably transduce MCF7 cells, according to the manufacturer's protocol (in the presence of 5 μg/ml polybrene). Twenty-four hours post-infection, media containing the virus was removed and replaced with standard media. Cells were then selected with puromycin (2 μg/ml), for up to 10 days. Please note that for most of the experiments described in this paper, we compared the GFP-high (top 5%) fraction to the GFP-low/negative (bottom 5%) fraction, unless stated otherwise. However, for label-free proteomics analysis, we compared the GFP-high (top 10%) population to the GFP-low (bottom 10%) population, to insure that enough material was collected for sample processing. For these experiments, singlet FACS gating of live cells was utilized.

### Mammosphere formation

A single cell suspension of MCF7-hTERT-eGFP cells was prepared using enzymatic (1x Trypsin-EDTA, Sigma Aldrich, #T3924), and manual disaggregation (25 gauge needle) [34]. Cells were then plated at a density of 500 cells/cm^2^ in mammosphere medium (DMEM-F12/B27/20-ng/ml EGF/PenStrep) in non-adherent conditions, in culture dishes coated with (2-hydroxyethylmethacrylate) (poly-HEMA, Sigma, #P3932). Cells were grown for 5 days and maintained in a humidified incubator at 37°C at an atmospheric pressure in 5% (v/v) carbon dioxide/air. After 5 days for culture, spheres >50 μm were counted using an eye piece graticule, and the percentage of cells plated which formed spheres was calculated and is referred to as percentage mammosphere formation, and was normalized to one (1 = 100% MSE). Mammosphere assays were performed in triplicate and repeated three times independently.

### Treatment with telomerase inhibitor (MST-312)

Cells were treated on day 0 with MST-312 at a concentration of 10 μM and compared to vehicle alone controls, processed in parallel, after 5 days of mammosphere culture. A dose-response analysis of the effects of MST-312 on parental MCF7 cells was first performed to establish an effective concentration for the inhibition of mammosphere formation; our results showed that 1 μM had little or no effect, while 10 μM inhibited mammosphere formation by approximately 70% (data not shown). Therefore, experiments with eGFP fractionated MCF7 cells were carried out at 10 μM.

### Microscopy

Fluorescent imaging of MCF7 cells in adherent conditions and non-adherent spheroid culture was performed using a Leica SP8 multi-photon DM6000 microscope to detect GFP expression and bright field images.

### Proteomics analysis

Cell lysates were prepared for trypsin digestion by sequential reduction of disulphide bonds with TCEP and alkylation with MMTS [35]. Then, the peptides were extracted and prepared for LC-MS/MS. All LC-MS/MS analyses were performed on an LTQ Orbitrap XL mass spectrometer (Thermo Scientific, San Jose, CA) coupled to an Ultimate 3000 RSLCnano system (Thermo Scientific, formerly Dionex, The Netherlands). Xcalibur raw data files acquired on the LTQ-Orbitrap XL were directly imported into Progenesis LCMS software (Waters Corp., Milford, MA, formerly Non-linear dynamics, Newcastle upon Tyne, UK) for peak detection and alignment. Data were analyzed using the Mascot search engine. Five technical replicates were analyzed for each sample type. Statistical analyses were performed using ANOVA and only fold-changes in proteins with a p-value less than 0.05 were considered significant.

### Bioinformatics analysis with human clinical samples

To firmly establish the clinical relevance of our results from the quantitative proteomics, we re-analyzed the transcriptional profiles of epithelial breast cancer cells and adjacent tumor stromal cells that were physically separated by laser-capture microdissection (from *N* = 28 human breast cancer patients) [10].

### FACS analysis

For live cell sorting experiments, hTERT-eGFP transfected MCF7 cells were resuspended in PBS and sorted according to eGFP expression using the BD influx. Untransfected MCF7 cells were used as a negative control to determine positive GFP expression. Cells were sorted into two groups, those with the highest GFP expression and those with the lowest GFP expression. For FACs analysis experiments, hTERT-eGFP cells were labeled with MitoTracker Deep Red (#M22426, Invitrogen) and MitoTracker Orange ((#M7510, Invitrogen). Cells were kept of ice until analysis using the FACS Calibur. Results were analyzed using FlowJo software version 10. Cells were gated into two populations, those with the highest 5% of GFP expression, and those with the lowest 5% and negative GFP expression. The median fluorescent intensity of MitoTracker Deep Red (640nm) and MitoTracker Orange (561nm) was then determined within the two distinct GFP cellular populations. Additionally, cells were discriminated by cell size into two populations, small and large using SSC (side scatter) and FSC (forward scatter). The median intensity of eGFP, MitoTracker Deep Red, and MitoTracker Orange, within these populations, was then determined.

### Mitochondrial staining

To measure mitochondrial activity, cells were stained with MitoTracker Orange (#M7510, Invitrogen), whose accumulation in mitochondria is dependent upon membrane potential. To measure mitochondrial mass, cells were stained with MitoTracker Deep Red (#M22426, Invitrogen), localizing to mitochondria regardless of mitochondrial membrane potential. Cells were incubated with pre-warmed MitoTracker staining solution (diluted in PBS/CM to a final concentration of 10 nM) for 30-60 min at 37 °C. All subsequent steps were performed in the dark. Cells were washed in PBS, harvested, and re-suspended in 300 μL of PBS. Cells were then analyzed by flow cytometry. Data analysis was performed using FlowJo software.

### Fractionation of MCF7 by cell size

hTERT-eGFP-MCF7 cells, co-labeled with MitoTracker dyes, were analyzed by cell size using FlowJo software. Cells were separated by gating of forward scatter (FSC) and side scatter (SSC) plots into two populations for analysis, large cells (∼15% of the total population) and small cells (∼85% of the total population). The median fluorescent intensity of eGFP and MitoTracker dyes were determined in each population of cells. Very similar results were obtained, with either singlet FACS gating or all live cell FACS gating.

### Statistical analyses

Statistical significance was determined using the Student's t-test or ANOVA, where appropriate. Values of less than 0.05 were considered significant. Data are shown as the mean ± SEM, unless stated otherwise.

## Abbreviations

CSCs: cancer stem-like cells
TICs: tumor-initiating cells
